# Super-Resolution Network with Information Distillation and Multi-Scale Attention for Medical CT Image

**DOI:** 10.3390/s21206870

**Published:** 2021-10-16

**Authors:** Tianliu Zhao, Lei Hu, Yongmei Zhang, Jianying Fang

**Affiliations:** 1 School of Computer Information Engineering, Jiangxi Normal University, Nanchang 330022, China; tianliuzhao@jxnu.edu.cn (T.Z.); jyfang@jxnu.edu.cn (J.F.); 2 School of Information Science and Technology, North China University of Technology, Beijing 100144, China; zhangym@ncut.edu.cn

**Keywords:** super-resolution, medical CT image, multi-scale attention, information distillation, deep learning

## Abstract

The CT image is an important reference for clinical diagnosis. However, due to the external influence and equipment limitation in the imaging, the CT image often has problems such as blurring, a lack of detail and unclear edges, which affect the subsequent diagnosis. In order to obtain high-quality medical CT images, we propose an information distillation and multi-scale attention network (IDMAN) for medical CT image super-resolution reconstruction. In a deep residual network, instead of only adding the convolution layer repeatedly, we introduce information distillation to make full use of the feature information. In addition, in order to better capture information and focus on more important features, we use a multi-scale attention block with multiple branches, which can automatically generate weights to adjust the network. Through these improvements, our model effectively solves the problems of insufficient feature utilization and single attention source, improves the learning ability and expression ability, and thus can reconstruct the higher quality medical CT image. We conduct a series of experiments; the results show that our method outperforms the previous algorithms and has a better performance of medical CT image reconstruction in the objective evaluation and visual effect.

## 1. Introduction

The computed tomography (CT) image is an important auxiliary means in clinical diagnosis. The image quality has a very significant impact on the diagnosis of lesions. High-quality medical images can help doctors to identify the symptoms more accurately and quickly formulate the corresponding treatment plan for patients. However, the limitation of imaging devices makes it difficult to obtain high-resolution medical CT images, so these images always have some problems such as low resolution, blurring and loss of detail. As a classic computer vision task, super-resolution (SR) reconstruction can use low-resolution (LR) images to reconstruct high-resolution (HR) images. Super-resolution algorithms can also be used in medical CT image to improve the image quality.

According to the different objects of SR processing, we can divide the super-resolution technique into single image super-resolution (SISR), multiple image super-resolution (MISR) and video super-resolution (VSR). Among them, SISR only uses one image to improve the resolution of the image. The requirement of input is relatively low, so there are many studies regarding SISR. SISR is one of the key research directions on image super-resolution. With the improvement performance of the super-resolution algorithm, the super-resolution network is gradually applied in the field of medical image. In this paper, we mainly discussed the single image super-resolution reconstruction for the medical CT image.

The existing super-resolution algorithms can be divided into three types according to the different implementation principles: the interpolation-based method [1,2], reconstruction-based method [3] and learning-based method [4,5,6]. In the early stage, the interpolation-based method and reconstruction-based method were the main methods. The method based on interpolation, such as the nearest neighbor interpolation and bicubic interpolation, is to calculate the new pixel value by calculating the weighted average value of the pixels around a certain pixel in the low-resolution image, and insert the new pixel value into the image to reconstruct the high-resolution image. The method, based on the reconstruction, uses the degradation model and prior knowledge to constrain the possible solution space of the image, and realize the reconstruction from the low-resolution image to the high-resolution image. However, these two methods have some disadvantages: they do not make full use of the image information and have a poor ability to restore high-frequency details.

In recent years, with the wide application of deep neural networks, Dong et al. proposed SRCNN [6] to introduce the convolutional neural network (CNN) to the image super-resolution task for the first time and achieved great success. From a low-resolution to a high-resolution image, the end-to-end mapping is realized by patch extraction and representation, non-linear mapping and reconstruction. Based on CNN, subsequently, scholars have proposed a series of super-resolution reconstruction algorithms and achieved advanced results. To accelerate the SRCNN, FSRCNN [7] was proposed in 2016, it performed upsampling at the end of the network and used an hourglass-shape CNN structure to reduce the computational cost. In the SRResNet [8] proposed by Ledig et al., residual learning was introduced to solve the problem of deep network training difficultly. Kim et al. [9] introduced recursive learning to increase the depth of the model without increasing the extra parameters. EDSR [10] significantly improved network performance and won the NTIRE2017 championship by removing redundant modules and expanding the model. For SR tasks, the success of EDSR also demonstrated the effectiveness of deepening the network. On this basis, Zhang et al. proposed the RCAN [11] with a residual in residual (RIR) structure to construct a very deep trainable network. It combined the residual structure with the attention mechanism and achieved further success. It turns out that the deep network with a residual structure and an attention block can achieve better results, so we attempt to apply it to reconstruct medical CT images. However, there are still some problems in the process when the current methods are applied to the medical image super-resolution task: most of the networks deepen the network by the repeated convolution operation but neglect the full use of the feature information. As a result, LR loses some information in the deep network transmission, and the information is not fully utilized. Another concern is the attention mechanism, which involves a single branch for general channel attention, resulting in a relatively single source of the characteristic information concerned by the network.

Inspired by the work in [11,12], we propose an information distillation and multi-scale attention network (IDMAN) to reconstruct the medical CT image by better learning the feature information. To solve the problem of insufficient information utilization, IDMAN uses information distillation to extract features step-by-step. Compared with the traditional operation of repeating the convolution layer, information distillation can effectively improve the learning ability of the network. Because the successes of EDSR and RCAN prove that the deep network has a remarkable ability to improve the performance of super-resolution network, IDMAN chooses the deep network and exploits the residual learning and the skip connection to make the network trainable. To change the single feature information source, IDMAN adopts the multi-scale attention with multiple branches to strengthen the attention to important features, and to improve the network expression ability of important features.

In summary, the main contributions of this paper are as follows:

- We propose an information distillation and multi-scale attention network for medical CT image super-resolution reconstruction, which can reconstruct sharper edges and more realistic textures, and restore more details of the CT image;
- In order to make full use of the feature information of the image, our proposed method combines information distillation with a deep residual network, so that the model can learn the image feature in a deep network, and distill the feature information instead of simply repeating the convolution operation;
- We use a multi-scale attention module with multiple branches to adaptively focus on the interdependence of feature channels from different branches and pay more attention to the important information of the CT image by assigning weights to the different features.

The rest of this paper is organized as follows. The related works are represented in Section 2. The proposed method of information distillation and multi-scale attention network is introduced in Section 3. We show the experimental results, model analysis and ablation studies in Section 4. The conclusions of this paper are stated in Section 5.

## 2. Related Works

In recent years, with the development of neural networks, super-resolution algorithms based on deep learning have become the main research focus of image processing. In order to achieve better results, the network was continuously widened and deepened. However, simply widening and deepening the network did not achieve the expected significant improvement. Therefore, scholars designed some exquisite network structures and learning strategies such as residual structures, dense connections, attention mechanisms, information distillation, transfer learning and so on. In this paper, our super-resolution reconstruction network for medical CT image references the residual network, attention mechanism and information distillation. The following section mainly describes the four aspects involved in this model for the current work: single image super-resolution, residual network, attention mechanism and information distillation.

### 2.1. Single Image Super-Resolution

Single image super-resolution is the basis of multiple image super-resolution and video super-resolution. Since SRCNN first applied the convolutional neural network to the SR task, a number of methods based on deep CNN have been proposed. Based on SRCNN, VDSR [13] used a deeper convolutional network and achieved better results. In order to reduce the complexity of the model, DRCN [9] proposed a deep recursive convolution network for SR task, which did not introduce new parameters but improved the performance of network. On the basis of DRCN, DRRN [14] proposed deep recursive residual network by combining residual learning and recursive learning. The sub-pixel convolution in ESPCN [15] was proposed by Shi et al. to extract features directly from low-resolution images and to upscale the image in the sub-pixel convolution layer. MemNet [16] proposed long-term memory networks to help build long-term dependence. LapSRN [17] made use of the Laplacian pyramid structure and achieved good results with the large upscaling factor through progressive reconstruction. SRDenseNet [18] introduced dense connections [19] in the deep networks, combining the low-level features and high-level features to improve reconstruction performance. EDSR [10] was optimized by removing unnecessary modules in the residual network and expanded the model size to further improve the performance and obtain good results. With the deepening of the network, the features of each convolutional layer would have different receptive fields. In order to make greater use of the information of each convolutional layer, Zhang et al. put forward the RDN [20] to make better use of the hierarchical information from the LR image. RCAN [11] combined the attention mechanism and the residual module to improve the network expression ability, achieving excellent results.

### 2.2. Residual Network

With the development of neural networks, their depth increased, and deep networks achieve better effects than shallow networks. However, with the deepening of the networks, gradient vanishing, gradient explosion and network degradation may occur. Therefore, He et al. [21] proposed a residual network (ResNet) for image classification, which effectively solves the problem of deep network training. The main characteristic of the residual structure is adding a skip connection on the basis of the convolutional neural network so that the original input information can be directly connected to the back layer, which protects the integrity of the original input information to a greater extent. The residual structure was introduced into the super-resolution reconstruction network in [8]. The subsequent studies of [10,11] deepened the network based on the residual block and achieved better results. By adding the residual structure, the deep network can effectively alleviate the problems of gradient vanishing and network degradation, speed up the training process, and further improve the network performance.

### 2.3. Attention Mechanism

The attention mechanism can help the network focus on local information, by constantly adjusting the weight, with a higher weight being more attentive to important information. Google DeepMind [22] combined the RNN model with the attention mechanism to classify images. The attention mechanism can be divided into spatial attention, channel attention and mixed attention. The spatial transformer network (STN) [23] proposed by Jaderberg et al. used the spatial attention which completed the processing operation suitable for the task by learning the deformation of the input. In 2017, the SENet [24] proposed by Hu et al. used the channel attention mechanism to assign different attention values to different channels through squeeze and excitation operations, which were used to increase the weight of the important channels and achieve the goal of paying more attention to the important feature channels. Then, the convolutional block attention module (CBAM) [25] proposed by Woo et al. combined spatial attention and channel attention, realized the fusion of different attention mechanisms and achieved good results. In addition, with the efforts of researchers, the attention mechanism also developed into self-attention, multi-scale attention, residual attention and recurrent attention, etc.

### 2.4. Information Distillation

Information distillation can enable the network to make greater use of the feature information of images by gradually extracting the input features. Hui et al. [26] proposed an information distillation network (IDN) to effectively extract the local long and short-path features by combing the enhancement unit with compression unit. Inspired by IDN, Hui et al. proposed the information multi-distillation network (IMDN) [27] to extract the features step-by-step. In the dimension of the channel, the feature was divided into two parts; one part was retained, the other part conducted a further distillation operation, then the two features were fused to obtain more information. Jie et al. put forward the residual feature distillation network (RFDN) [12] by combining the information distillation with residual learning.

## 3. Methods

### 3.1. Network Architecture

The proposed IDMAN combines information distillation and multi-scale attention to extract deep features and uses the local feedback for the fusion of the features to reconstruct the CT image. If the network expectations improve the learning ability only by repeatedly adding many convolution layers, a significant performance improvement may not be achieved. In order to make full use of the feature information, information distillation is introduced into the deep network, and the feature can be better distilled and refined. In addition, the information distribution of the CT image is not uniform. With the purpose of paying more attention to important information, we also utilized the multi-scale attention block (MAB). On the basis of progressively extracting the refined features, multi-scale attention is used to concern the important feature information under different branches, so that the network can capture more details and reconstruct higher quality images.

**Figure 1:**
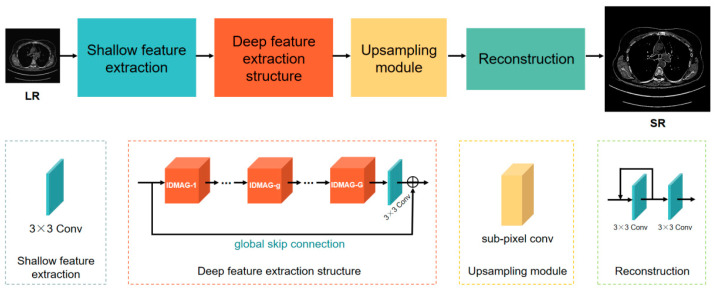
The architecture of information distillation and multi-scale attention network (IDMAN) comprises four parts: shallow feature extraction, deep feature extraction structure (DFES), upsampling module and reconstruction.

As shown in Figure 1, the IDMAN consists of four parts: the shallow feature extraction, deep feature extraction structure (DFES), upsampling module and reconstruction. Firstly, one convolution layer is used to extract the shallow features. Then, the deep features are extracted through DFES, which contains G information distillation and multi-scale attention groups (the details are discussed in Section 3.2.), one convolution layer and a global skip connection. Next, the upsampling module is used to enlarge the image. Finally, the output of the upsampling module is used to reconstruct the image by a local feedback and one convolution layer, and the reconstructed super-resolution image is obtained.

Define $I_{LR}$ and $I_{SR}$ as the input and output of IDMAN. We extract the shallow feature $F_{0}$ from $I_{LR}$ by using one convolution layer from the *LR* input:

(1) $$F_{0}=H_{SFE}\left(I_{LR}\right)$$

where $H_{SFE}\left(\cdot \right)$ represents the 3×3 convolution operation. After obtaining the shallow feature $F_{0}$, we feed it into the *DFES*, which is used to extract the deep feature. We can obtain the deep feature $F_{DF}$:

(2) $$F_{DF}=H_{DFES}\left(F_{0}\right)$$

where $H_{DFES}\left(\cdot \right)$ is the deep feature extraction structure (*DFES*) to extract the deep feature. The deep feature $F_{DF}$ is input into the upsampling module:

(3) $$F_{UP}=H_{UP}\left(F_{DF}\right)$$

where $H_{UP}\left(\cdot \right)$ denotes the upsampling module. We use the sub-pixel convolution [15] for upsampling. The upscaled feature $F_{UP}$ is reconstructed as follows:

(4) $$I_{SR}=H_{REC}\left(F_{UP}\right)$$

where $H_{REC}\left(\cdot \right)$ denotes the reconstruction operation. Through local feedback operation on the convolution, $F_{UP}$ is better reconstructed without increasing the extra parameters. Finally, we can obtain the reconstructed image $I_{SR}$.

The whole network can be described as:

(5) $$I_{SR}=H_{IDMAN}\left(I_{LR}\right)$$

where $H_{IDMAN}\left(\cdot \right)$ denotes the *IDMAN*.

Using $L_{1}$ loss function [28] to optimize the network, as in previous works, for the training set ${\left\{I_{LR}^{i},I_{HR}^{i}\right\}}_{i=1}^{N}$, which contains *N LR* inputs and their *HR* counterparts, the goal of training *IDMAN* is to minimize $L_{1}$ loss function:

(6) $$L\left(\theta \right)=\frac{1}{N}\overset{N}{\underset{i=1}{\sum }}∥H_{IDMAN}\left(I_{LR}^{i}\right)-I_{HR}^{i}{∥}_{1}$$

where θ denotes the parameter set of the network. The loss function is optimized by the stochastic gradient descent method.

### 3.2. Information Distillation and Multi-Scale Attention Group

The network extracts the deep features through the DFES. DFES is mainly composed of G information distillation and multi-scale attention groups (IDMAG). For the input feature $F_{in}$, the *g*-th IDMAG in DFES can be expressed as:

(7) $$F_{g}=H_{g}\left(F_{g-1}\right)=H_{g}\left(H_{g-1}\left(\cdots H_{1}\left(F_{in}\right)\cdots \right)\right)$$

where $H_{g}\left(\cdot \right)$ denotes the function of *g*-th IDMAG, $F_{g-1}$ and $F_{g}$ are the input and output of the *g*-th IDMAG. After G IDMAG, one convolution layer is used to fuse features and a global skip connection is used to ensure the shallow feature is not lost:

(8) $$F_{G\_out}=F_{in}+H_{conv}\left(F_{G}\right)$$

where $H_{conv}\left(\cdot \right)$ denotes the 3×3 convolution operation, $F_{G}$ denotes the output of *G*-th IDMAG and $F_{G\_out}$ denotes the output of the DFES, which includes *G* IDMAG.

**Figure 2:**
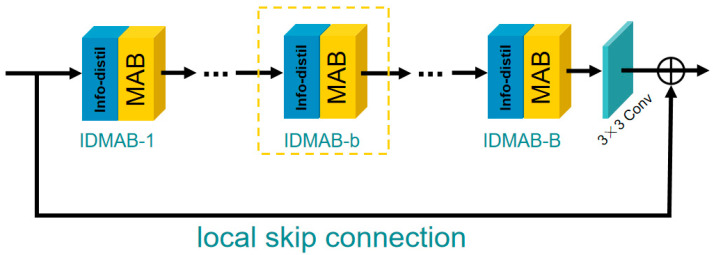
Information distillation and multi-scale attention group (IDMAG).

The structure of IDMAG is shown in Figure 2. Similar to DFES, the IDMAG consists of B information distillation and multi-scale attention blocks (IDMAB) (more details about IDMAB would be discussed in Section 3.3), one 3×3 convolution layer and a local skip connection.

In the *g*-th IDMAG, the output through the b IDMAB blocks can be expressed as:

(9) $$F_{g,b}=H_{g,b}\left(F_{g,\;b-1}\right)=H_{g,b}\left(H_{g,\;b-1}\left(\cdots H_{g,1}\left(F_{g-1}\right)\cdots \right)\right)\;F_{\left(G,\;B\right)\_out}=F_{G-1}+H_{conv}\left(F_{G,\;B}\right)$$

where $H_{g,b}\left(\cdot \right)$ denotes the operation to extract the features of the *b*-th IDMAB in the *g*-th IDMAG, $F_{g-1}$ denotes the output of the (*g −* 1)-th IDMAG, $F_{g,b}$ denotes the output of the *b*-th IDMAB in the *g*-th IDMAG, $H_{conv}\left(\cdot \right)$ denotes the 3×3 convolution operation, $F_{\left(G,\;B\right)\_out}$ denotes the output of the G-th IDMAG which contains B IDMAB. Feature $F_{G,\;B}$ was obtained by using B IDMAB, then using a 3×3 convolution layer to fuse local features and add the local skip connection to solve the possible gradient vanishing problem.

### 3.3. Information Distillation and Multi-Scale Attention Block

**Figure 3:**
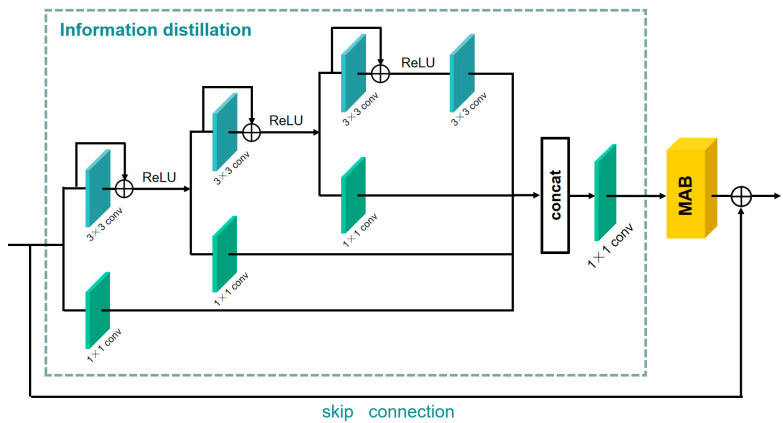
Information distillation and multi-scale attention block (IDMAB).

As shown in Figure 3, the information distillation and multi-scale attention block (IDMAB) consists of information distillation, multi-scale attention block (MAB) and a skip connection.

#### 3.3.1. Information Distillation

For the input feature $f_{in}$ of IDMAB, the information distillation first goes through three refining distillation steps. For each step, the preceding feature is divided into two parts through a channel splitting operation. One part is preserved by a 1×1 convolution layer. The other part further extracts the feature by the 3×3 convolution layer and residual learning, then the feature is activated by the ReLU operation and transported to the next distillation step. Three features ($f_{distilled\_1}$, $f_{distilled\_2}$, $f_{distilled\_3}$) retained in the distillation process, and one feature ($f_{distilled\_4}$) further extracted by the 3×3 convolution layer is obtained after distillation steps. All features obtained after distillation are concatenated together to obtain the feature $f_{distilled}$, then a 1×1 convolution layer is adopted to further fuse the feature. This structure can be described as:

(10) $$f_{distilled\_1},\;f_{coarse\_1}=H_{distilled\_1}\left(f_{in}\right),H_{coarse\_1}\left(f_{in}\right)\;f_{distilled\_2},\;f_{coarse\_2}=H_{distilled\_2}\left(f_{coarse\_1}\right),H_{coarse\_2}\left(f_{coarse\_1}\right)\;f_{distilled\_3},\;f_{coarse\_3}=H_{distilled\_3}\left(f_{coarse\_2}\right),H_{coarse\_3}\left(f_{coarse\_2}\right)\;f_{distilled\_4}=H_{conv}\left(f_{coarse\_3}\right)$$

where $H_{distilled\_i}\left(\cdot \right)$ is a 1×1 convolution operation which can produce the retained feature in *i*-th step of distillation. The $H_{coarse\_i}\left(\cdot \right)$ denotes the further refinement of the coarse features in *i*-th step of distillation, which consists of a 3×3 convolution layer, identity connection, and the activation unit (ReLU). $H_{conv}\left(\cdot \right)$ denotes the 3×3 convolution operation. $f_{distilled\_i}$ denotes the *i*-th distilled feature (preserved), and the $f_{coarse\_i}$ denotes the *i*-th coarse feature that requires further processing. Then, all the distilled features are fused:

(11) $$f_{distilled}=H_{fuse}\left(Concat\left(f_{distilled_{1}},f_{distilled_{2}},f_{distilled_{3}},f_{distilled\_4}\right)\right)$$

where Concat(·) denotes concatenation operation among the channel dimension, $H_{fuse}\left(\cdot \right)$ denotes the fusion operation, which uses the 1×1 convolution layer to fuse the features obtained by distillation. Then, the aggregated feature $f_{distilled}$ is fed into the *MAB*, and the output of IDMAB is obtained:

(12) $$f_{out}=f_{in}+H_{MAB}\left(f_{distilled}\right)$$

where $H_{MAB}\left(\cdot \right)$ means the feature extraction using a multi-scale attention block (*MAB*), $f_{out}$ denotes the output of IDMAB.

#### 3.3.2. Multi-Scale Attention Block

In order to pay more attention to the important features and make the network adaptively allocate the weight according to the importance of different information, we also replace the channel attention with the multi-scale attention block (MAB). The multiple branches of MAB can make the network focus on more abundant feature information and the interdependencies among feature channels, and make the model pay different degrees of attention according to the different channel importance.

**Figure 4:**
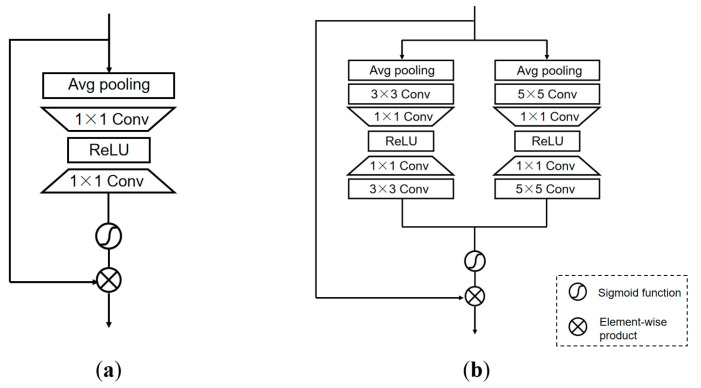
Different types of attention module: (**a**) Channel attention; (**b**) Multi-scale Attention block (MAB).

Compared to the traditional channel attention module (Figure 4a), the multiple-branch structure of the MAB (Figure 4b), with 3×3 and 5×5 branches, can better capture the information because of the feature fusion of the two branches. In the 3×3 branch, we first use the average pooling to compress spatial information, and next adopt a 3×3 convolution layer to extract the feature information. Then, we use a 1×1 convolution layer to compress the channel and adopt ReLU to activate the result. Subsequently, we reuse the 1×1 convolution layer to increase the low-dimension information and extract the feature again by a 3×3 convolution layer. Refer to the branch 3×3, the branch 5×5 has similar operations. At the end of MAB, the features from the two branches are fused, and the result has multi-scale feature information.

In each branch of MAB, the channel-wise spatial information is taken into the channel descriptors through average pooling. Let X be the input, then the channel-wise statistic z can be obtained after average pooling:

(13) $$z=H_{AP}\left(X\right)$$

where $H_{AP}\left(\cdot \right)$ is the pooling function.

For the MAB with *n* branches, the convolution kernel size of each branch is different. Each convolution operation with a different kernel size can be described as:

(14) $$z^{n,k_{n}}=h_{n,k_{n}}\left(z\right)$$

where $h_{n,k_{n}}\left(\cdot \right)$ is the convolution operation with the kernel size $k_{n}\times k_{n}$ in the *n*-th branch. In this paper, we set n=1, 2 and $k_{1}=3,\;k_{2}=5$.

In order to fully use of the interdependence of features and establish the correlation between the channels, the sigmoid function is used as a gating mechanism to learn the interactions between the channels and control the aggregated information generated by the pooling operation. Before that, we need to process each branch as follows:

(15) $$\omega_{n,k_{n}}=h_{n,k_{n}}\left(W_{U}\cdot \delta \left(W_{D}\cdot z^{n,k_{n}}\right)\right)$$

where δ(·) denotes the ReLU function and $h_{n,k_{n}}\left(\cdot \right)$ denotes the convolution operation with the kernel size $k_{n}\times k_{n}$ in the *n*-th branch. $W_{U}$ and $W_{D}$ are the weight sets from 1×1 convolution layer, respectively. $W_{D}$ is the weight set of the 1×1 channel-downsampling convolution layer by a reduction ratio r. The low-dimension information is activated with ReLU and then increased again with the ratio r. $W_{U}$ is the weight set of the 1×1 channel-upsampling convolution layer. $\omega_{n,k_{n}}$ denotes the channel statistics in the *n*-th branch with a kernel size of $k_{n}\times k_{n}$. The statistics of the branches are added to the total channel statistics ω:

(16) $$\omega =f\left(\omega_{1,k_{1}}+\cdots +\omega_{n,k_{n}}\right)$$

where f(·) denotes the sigmoid gating. MAB in this paper consists of two branches; the kernel size of convolution is 3×3 and 5×5, respectively, so $\omega =f\left(\omega_{1,k_{1}=3}+\omega_{2,k_{2}=5}\right)$. Additionally, the obtained channel statistics ω are used to rescale the input X:

(17) $$\hat{X}=\omega \cdot X$$

where ω and X are the scaling factors and feature maps, respectively.

## 4. Results

In this section, we describe the dataset and the implementation details of the model. Two common image quality evaluation indexes PSNR and SSIM are adopted to evaluate the model objectively. We also compare our model with other advanced super-resolution methods and analyze the results.

### 4.1. Dataset

The experiment was conducted using DeepLesion [29], at present the largest dataset of CT medical images in the world. We randomly selected 11,500 high-quality CT images, of which 10,000 were used for training, 500 for validating and 1000 for testing. The dataset needed to be preprocessed, which was achieved through downsampling by bicubic interpolation through MATLAB R2017b. In this way, the HR images were degraded into the LR images to form the image pair for training. Then, we augmented the training set, which was randomly rotated by 90°, 180°, 270° and flipped horizontally to improve the generalization ability. For the testing sets, we also used two public medical image datasets, NSCLC Radiogenomics and Lung-PET-CT-Dx from TCIA [30], and randomly selected 500 medical images from each of these two datasets for testing.

### 4.2. Implementation Details

In our method, the number of IDMAG is set to 10 and the number of IDMAB is set to 20. Because of the GPU limitations, the batch size is set to 10. We take the LR image and corresponding HR image as the input and crop the patch with the size of 48×48, and the reduction ratio r of MAB is set to 16. We choose the L1 loss function and use the ADAM Optimizer [31], with $\beta_{1}=0.9,{\;\beta }_{2}=0.999,\;\epsilon =10^{-8}$. The initial learning rate is set to $10^{-4}$, and the learning rate is decreased by half every $2\times 10^{5}$ iterations. Most of our parameters refer to RCAN [11]. We set the training parameters of the contrast algorithms (such as batch size and learning rate) as consistent with our method, to compare the performance of different network architectures.

Our method is implemented using pytorch 0.4 and Python 3.6 in the Ubuntu 18.04 operating system, with an Inter E5-2620 CPU and an Nvidia GTX 1080TI GPU.

### 4.3. Evaluation Indexes

In order to verify the performance of the super-resolution reconstruction network, it is necessary to evaluate the reconstructed image. There are two methods to evaluate image quality: objective evaluation and subjective evaluation. Subjective evaluation is affected by many aspects, it mainly evaluates the reconstructed SR image from the visual effect. The objective evaluation is to quantitatively analyze the image and evaluate it through specific evaluation indicators. In this paper, the performance of super-resolution networks is evaluated by using two recognized image quality indicators, the Peak Signal-to-Noise Ratio (PSNR) [32] and Structure Similarity Index (SSIM) [33]. The PSNR and SSIM of the reconstructed SR results are calculated by the MATLAB to compare our method and other methods.

PSNR. *PSNR* (dB) is based on the error between the corresponding pixels of the image pair. It is an objective standard for evaluating the image. The unit of *PSNR* is dB. In general, the higher *PSNR* represents a higher resolution image. It is defined by *MSE*:

(18) $$MSE=\frac{1}{mn}\overset{m-1}{\underset{i=0}{\sum }}\overset{n-1}{\underset{j=0}{\sum }}{\left|\left|I\left(i,j\right)-K\left(i,j\right)\right|\right|}^{2}$$

Then, *PSNR* can be expressed as:

(19) $$PSNR=10{log}_{10}\frac{MaxValue^{2}}{MSE}=10log_{10}\frac{2^{bits}-1}{MSE}$$

where I and K are represented as images with a size of m×n. The larger the *PSNR*, the smaller the image distortion and the better the image quality.

SSIM. *SSIM* is an index used to measure the structural similarity between two images, which measures the image similarity from the luminance, contrast and structure. The *SSIM* expression is:

(20) $$SSIM=\frac{\left(2\mu_{x}\mu_{y}+C_{1}\right)\left(2\sigma_{xy}+C_{2}\right)}{\left(\mu_{x}^{2}+\mu_{y}^{2}+C_{1}\right)\left(\sigma_{x}^{2}+\sigma_{y}^{2}+C_{2}\right)}$$

where $\mu_{x}$ represents the average value of image x, $\mu_{y}$ represents the average of image y, $\sigma_{x}$ represents the variance of image x, $\sigma_{y}$ represents the variance of image y, $\sigma_{xy}$ represents the covariance of x and y, and $C_{1}$ and $C_{2}$ are constants. The higher the *SSIM* value, the more similar the reconstructed image is to the original image, and the better the image quality.

### 4.4. Ablation Studies

This section mainly verifies the influence of some modules and strategies on our CT image reconstruction network. Among them, in order to prove the effect of information distillation and MAB on the model, we retain or remove the corresponding module to conduct the following ablation experiments.

The effectiveness of information distillation. IDMAN extracts the feature through information distillation (info-distill) to effectively utilize and learn feature information. In Table 1, Conv means extract the feature by using traditional convolution (Conv-ReLU-Conv), and Info-distill means to extract the feature by using information distillation. In order to avoid the influence of MAB, the attention mechanism adopts the traditional channel attention. As shown in Table 1, it can be seen that, compared with the traditional convolution operation, using information distillation to extract features can effectively improve PSNR, which means that our method, which uses info-distill, can make more use of feature information.

**Figure 5:**
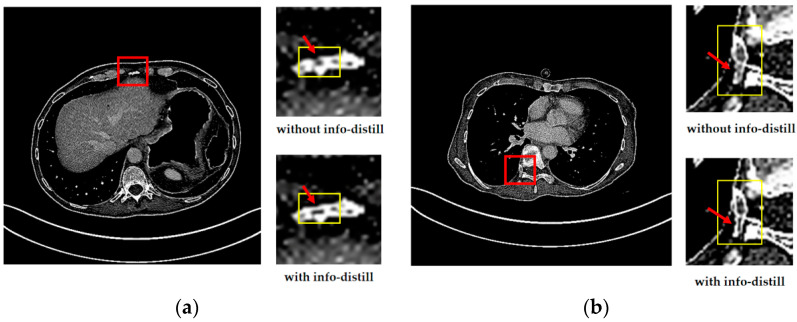
The reconstructed results of IDMAN with/without information distillation. (**a**) A reconstructed image of clear edges; (**b**) A reconstructed image of accurate details.

To show the reconstructed results more vividly and intuitively, we select two sets of images. We test the performance of IDMAN (with info-distill) and the model without info-distill. The reconstruction results are shown in Figure 5.

From Figure 5a, it easy to see that the edge of the reconstructed image is broken without info-distill, while the image can restore clearer edges when the information distillation is introduced. In Figure 5b, it is obvious that more accurate details can be reconstructed when using information distillation.

It can be seen that more details can be obtained in the reconstruction results with information distillation, which also proves that our method can make better use of feature information to improve learning ability when using information distillation to extract the feature. The improvements in both the subjective visual reconstruction results and objective evaluation index show that using information distillation to make more use of feature information is effective.

The effectiveness of MAB. In order to explore the influence of MAB with multiple branches, we designed several sets of contrasting experiments to verify whether single or multiple branches with different convolution kernel sizes could improve the network learning ability and expression ability by capturing information under different branches. The result on the validation set is shown in Table 2. The convolution kernel size 3×3, 5×5 and 7×7 corresponded to the branches of 3×3, 5×5 and 7×7. When none of them occurred, it meant the traditional channel attention was used (1×1-ReLU-1×1). In addition, to avoid the influence of information distillation, the traditional convolution operation (Conv-ReLU-Conv) was used for feature extraction.

We can draw some conclusions by analyzing the data from Table 2. First, under the single branch (Number of branches = 1), the performance could be improved by adding two convolution layers. For instance, the PSNR increased from 33.966 dB to 34.008 dB when we added two 3×3 convolution layers based on traditional channel attention. This demonstrated that the feature information could be better extracted by adding convolution layers. Second, it was obvious that, compared with the single branch MAB, the MAB with multiple branches (Number of branches is 2 or 3) could achieve a higher PSNR value. In other words, the performance of multiple branches was better than that of a single branch. The feature information could be better captured by our MAB with multiple branches structure.

The MAB with 3 × 3, 5 × 5, and 7 × 7 branches works better than the MAB with one or two branches. However, these comparison experiments are conducted without information distillation; if information distillation is introduced, the MAB can only have at most two branches, 3 × 3 and 5 × 5, due to the limitation of GPU. Therefore, our method selects MAB with 3 × 3 and 5 × 5 branches to better capture feature information as much as possible.

Table 3 shows the quantitative evaluation results of each module on the validation set. The experimental results show that PSNR are improved when adding the information distillation or MAB. When two modules are added at the same time, based on information distillation to the extract feature and the combination with MAB, the experimental results are the best (PSNR = 34.022 dB).

The effectiveness of weight normalization. EDSR improves the performance of the network by removing the batch normalization (BN). Inspired by this, we also adjust the normalization in the network. The weight normalization (WN) can accelerate the convergence of the deep learning network parameters by reparameterizing the weights of the network which decoupling the norm of the weight vector from the direction of the weight vector. Therefore, we normalize the convolution layer through WN in IDMAN.

Assume the output is y:

(21) y=wx+b

where x denotes the k-dimensional vector of the input features, w denotes the k-dimensional vector of weight, b denotes the scalar bias term. WN uses Formula (22) to re-parameterize the weight vector w:

(22) $$w=\frac{g}{\left||v|\right|}v$$

where g denotes the scalar, v denotes the k-dimensional vector, ||v|| denotes the Euclidean norm of v. Further, we can obtain ||w||=g, which is independent of the parameters v.

We have two experiments about WN. From Table 4, it can be seen that IDMAN with WN can get the higher PSNR value. This also can prove that WN has a positive effect on our reconstruction network.

**Figure 6:**
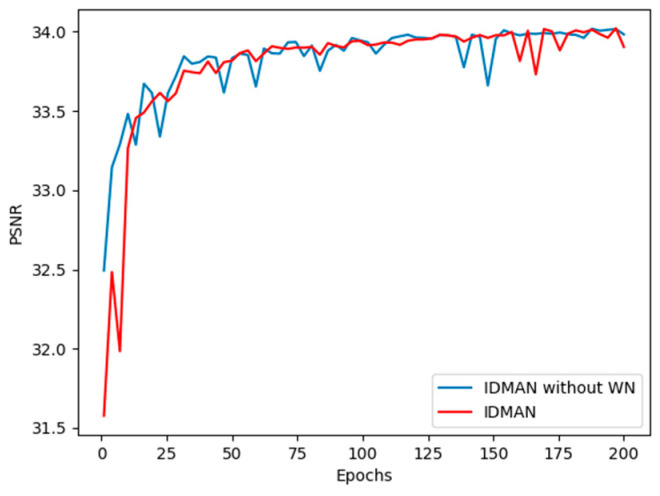
Convergence analysis of WN with scaling factor ×2.

In Figure 6, we can see that the curve of IDMAN without WN is steeper before 100 epochs, while the training of network with WN is more stable and the convergence speed is faster. Therefore, WN can accelerate the convergence of IDMAN and help the network to better learn the feature information.

### 4.5. Analysis of Experimental Results

Quantitative Analysis: In order to evaluate the performance of the model, we select some representative methods as the contrast. These methods are Bicubic [1], SRCNN [6], FSRCNN [7], VDSR [13], DRRN [14], EDSR [10], MDSR [10], RDN [20], and RCAN [11]. PSNR and SSIM are adopted to evaluate the quality of each SR result.

Table 5 and Table 6 summarize the PSNR and SSIM results of the quantitative evaluation of the scaling factors on ×2, ×3 and ×4. As shown from Table 5 and Table 6, compared with Bicubic, PSNR and SSIM improved by 8.601~11.683 dB and 17.98~20.63%, respectively. Compared with the methods based on deep learning, PSNR and SSIM improved by 0.063~1.92 dB and 0.06~0.98% when the scaling factor was ×2. Under scaling factor ×3, PSNR and SSIM increased by 0.026~2.081 dB and 0.01~1.44%. Under ×4, they increased by 0.013~2.562 dB and 0~2.46%, respectively. It is clear that the Bicubic reconstruction based on the traditional interpolation method is the worst, PSNR and SSIM are the lowest, while the SR algorithm based on deep learning is clearly better than that based on the interpolation method. In either case, the reconstruction effect of the low scaling factor is better than the high scaling factor. The PSNR and SSIM values show that the performance of the IDMAN is better than that of the comparative methods, which proves the superiority of our proposed method.

In order to compare more comprehensively and to further show the universality advantage of IDMAN on other datasets. We also tested our method under different scaling factors on the medical datasets NSCLC Radiogenomics and Lung-PET-CT-Dx.

Table 7 shows the testing results on the NSCLC Radiogenomics dataset. Under ×2 scale, compared with other methods, the improvements of IDMAN in PSNR and SSIM are 0.076~14.016 dB and 0.02~16.23%. Under the ×3 scale, the improvements are 0.05~11.598 dB and 0.02~17.36%. Additionally, under the ×4 scale, the improvements are 0.062~10.456 dB and 0.01~18.36%. Table 8 shows the results for the Lung-PET-CT-Dx dataset. The improvements of PSNR are 0.097~16.329 dB, 0.094~12.939 dB and 0.055~11.09 dB for ×2, ×3 and ×4 scaling factors. The improvements of SSIM are 0.01~14.46%, 0.03~16.5% and 0.02~18.45%, respectively. It can be seen that, for both testing sets, our IDMAN almost achieves the best performance with all scaling factors and improved to different degrees.

**Figure 7:**
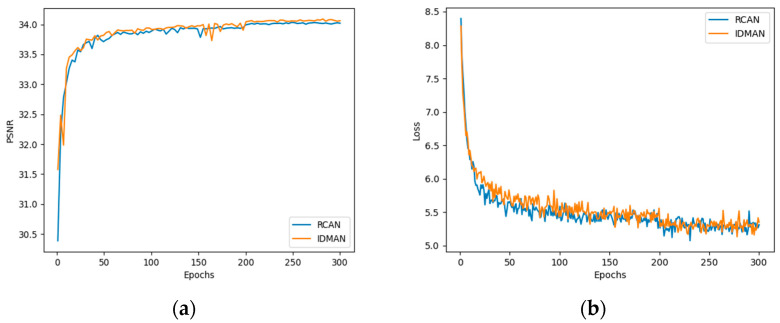
Convergence analysis with scaling factor ×2: (**a**) The PSNR curve during training; (**b**) The L1 loss curve during training.

Furthermore, we compared the convergence curves of our proposed IDMAN and suboptimal RCAN. As shown in Figure 7, we visualized the results of PSNR and L1 loss.

Figure 7a shows the curves of PSNR during training. It can be seen that the value of PSNR gradually stabilizes and no longer increases significantly during the training up to 200 to 300 epochs. Before approximately 200 epochs, the convergence speed of the IDMAN was slightly faster than RCAN, and the PSNR value of IDMAN mostly outperformed the other. As can be seen in Figure 7b, the loss shows an overall decreasing trend as the training epoch increases. Before about 100 epochs, the loss of RCAN decreases more rapidly, but after about 200 epochs, the loss of RCAN and IDMAN tend to become stable.

**Figure 8:**
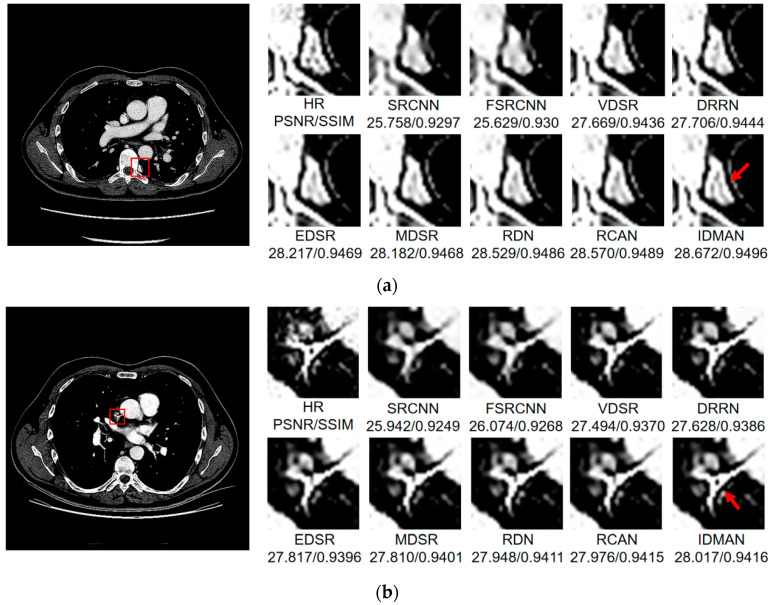

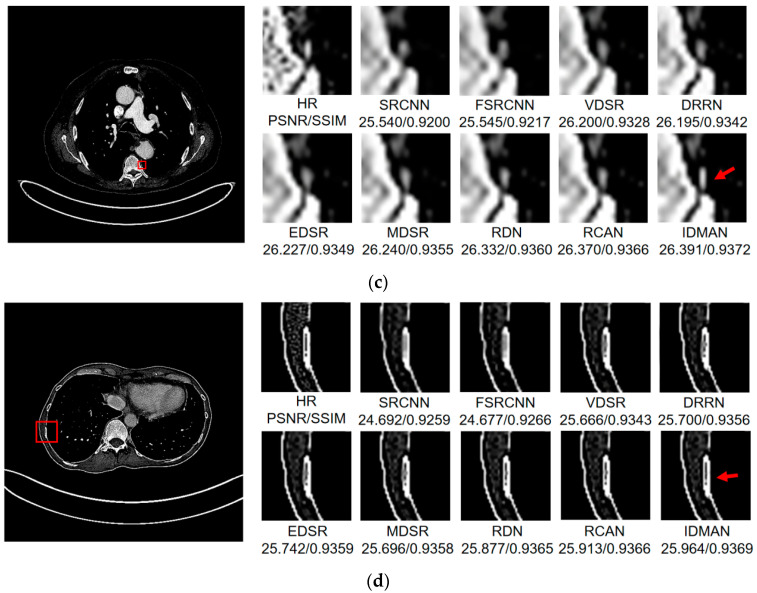
Images (**a**–**d**) show the visual results of different algorithms on DeepLesion testing set for scaling factor ×2.

**Figure 9:**
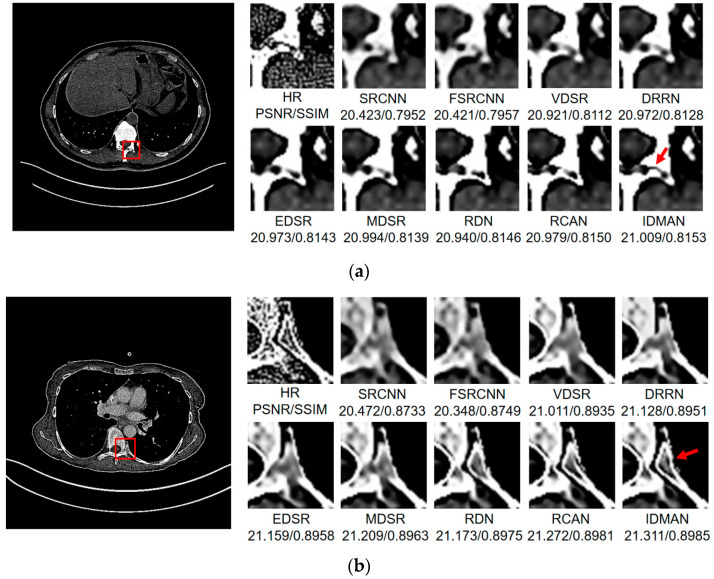
Images (**a**,**b**) show the visual results of different algorithms on DeepLesion testing set for scaling factor ×3.

**Figure 10:**
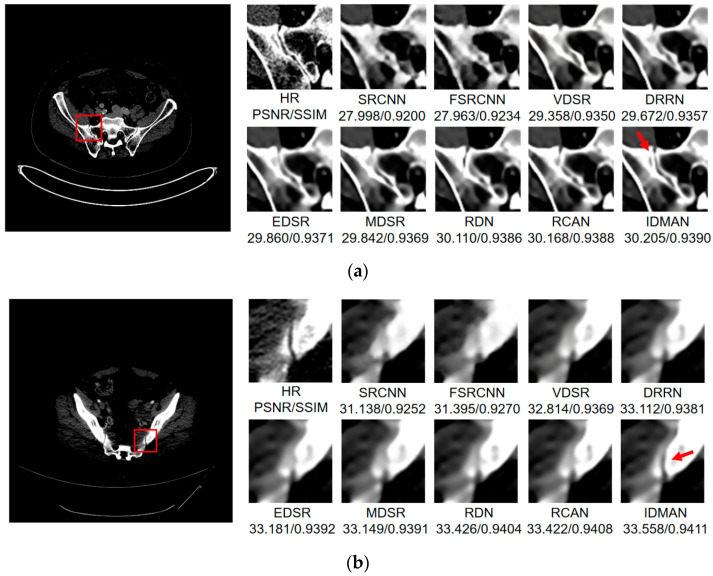
Images (**a**,**b**) show the visual results of different algorithms on DeepLesion testing set for scaling factor ×4.

Visual Results: In order to analyze the reconstructed CT image from the subjective visual effect, we select several groups of CT image for a contrast display in Figure 8, Figure 9 and Figure 10.

On the whole, The IDMAN proposed in this paper can reconstruct clearer edges and more realistic textures. Under the ×2 scale SR, as shown in Figure 8a, it is clear that the image reconstructed by IDMAN has a more realistic texture and a better visual effect. In Figure 8b, the reconstructed SR image can restore more truthful details than other methods. In Figure 8c, the image reconstructed by the contrast method is blurred, while the reconstructed result by our method has sharpener edges and more detail. In Figure 8d, the reconstructed image has a clearer and smoother edge. Under ×3 scale SR, from Figure 9a, it can be seen that the structure of the reconstructed image by other algorithms is more unclear, while the reconstructed result of IDMAN is more realistic. In Figure 9b, our reconstructed image has a more restored effect with clearer and more accurate contour lines, compared to other methods. Under the ×4 scale SR, we can observe, from Figure 10a, that the result of our method can better restore the original information and is more similar to the original image. In Figure 10b, it is clear that IDMAN can reconstruct a clearer edge but the images of other methods appear more blurred. Our proposed model achieved better results in the CT image dataset. The reconstructed CT image of our method has more details, and PSNR and SSIM also achieved higher scores.

It can be clearly observed that the reconstruction effect of SRCNN with only three layers is the worst among these methods based on deep learning, with problems such as blurring, artifacts, a lack of detail, unclear edges and so on. The later improved models are becoming more and more complex by deepening the network or using different learning strategies, and the reconstructed results have a clearer structure which is better than SRCNN. Additionally, our IDMAN has a superior learning ability, and the reconstructed results become to have more details and sharper edges. The better visual reconstruction results also prove that we can use information distillation and multi-scale attention to help the network make full use of the feature information more effectively, and capture more information to restore more details.

## 5. Conclusions

In this paper, we proposed an improved information distillation and multi-scale attention network for the medical CT image super-resolution, which combined the information distillation and multi-scale attention block. It effectively solved the problem of losing details, the insufficient use of feature information and the single branch of the attention block. We also conducted a series of experiments to prove the effectiveness of the IDMAN, and used ablation studies to show that information distillation and MAB had a positive effect on improving the network performance. We adopted PSNR and SSIM for the quantitative analysis, which were clear improvements compared with other methods. The reconstructed results had a clearer and more realistic edge and texture. In a word, the obtained results of the proposed IDMAN were better than those previous methods whether in objective evaluation indexes or in the subjective visual effect.

However, there are improvements that may benefit our work. Because the network is very deep, the parameter is very large and limited by the hardware, and the training time is relatively long. Additionally, the medical imaging is affected by the hardware equipment and the external environment which may produce the image noise. The next step is to balance the performance and training time, improve the training speed while maintaining the network performance, and effectively denoise the medical CT image while reconstructing it.

## Figures and Tables

**Table 1:** The effectiveness of information distillation of the model. We compared the traditional convolution and information distillation after 2 × 10^5^ iterations on the validation set for ×2 scale SR.

Scale	Conv	Info-Distill	PSNR
×2	√		33.966
		√	34.012

**Table 2:** The effectiveness of MAB with different branches on the model. We compared the results of our MAB with different branches after $2\times 10^{5}$ iterations on the validation set for ×2 scale SR.

MAB with Different Branches				
Number of Branches	3×3	5×5	7×7	PSNR
1				33.966
	√			34.008
		√		34.013
			√	34.006
2	√	√		34.014
	√		√	34.013
		√	√	34.016
3	√	√	√	34.018

**Table 3:** The quantitative results of each module. The best PSNR (dB) results on the validation set after $2\times 10^{5}$ iterations.

Different Combinations of Info-Distill, MAB (Scaling Factor ×2)				
Info-distill		√		√
MAB			√	√
PSNR	33.966	34.012	34.014	34.022

**Table 4:** The effectiveness of WN on the model. We compared the result of network without WN after $2\times 10^{5}$ iterations on the validation set for ×2 scale SR.

Scale	IDMAN without WN	IDMAN	PSNR
×2	√		34.019
		√	34.022

**Table 5:** The average PSNR (dB) results of different algorithms on the DeepLesion testing set with different scaling factors.

Model	×2	×3	×4
Bicubic	22.373	21.418	20.755
SRCNN	32.136	28.871	26.794
FSRCNN	32.162	28.880	26.900
VDSR	33.656	30.367	28.514
DRRN	33.709	30.485	28.670
EDSR	33.857	30.649	28.924
MDSR	33.858	30.666	28.937
RDN	33.973	30.869	29.255
RCAN	33.993	30.926	29.343
IDMAN (ours)	34.056	30.952	29.356

**Table 6:** The average SSIM results of different algorithms on the DeepLesion testing set with different scaling factors.

Model	×2	×3	×4
Bicubic	0.7649	0.7169	0.6832
SRCNN	0.9349	0.8940	0.8649
FSRCNN	0.9360	0.8955	0.8680
VDSR	0.9419	0.9051	0.8843
DRRN	0.9429	0.9056	0.8847
EDSR	0.9432	0.9067	0.8864
MDSR	0.9434	0.9067	0.8864
RDN	0.9441	0.9079	0.8889
RCAN	0.9441	0.9083	0.8895
IDMAN (ours)	0.9447	0.9084	0.8895

**Table 7:** The average PSNR and SSIM results of different algorithms in NSCLC Radiogenomics dataset with different scaling factors.

Model	×2		×3		×4	
	PSNR	SSIM	PSNR	SSIM	PSNR	SSIM
Bicubic	24.795	0.8132	24.060	0.7848	23.477	0.7643
SRCNN	36.924	0.9723	33.518	0.9517	31.599	0.9377
FSRCNN	37.152	0.9727	33.742	0.9525	31.746	0.9387
VDSR	38.232	0.9742	35.062	0.9567	32.728	0.9446
DRRN	38.362	0.9745	35.234	0.9571	33.285	0.9458
EDSR	38.587	0.9748	35.399	0.9574	33.586	0.9465
MDSR	38.590	0.9750	35.385	0.9573	33.481	0.9463
RDN	38.735	0.9753	35.504	0.9580	33.700	0.9473
RCAN	38.722	0.9753	35.608	0.9582	33.871	0.9478
IDMAN (ours)	38.811	0.9755	35.658	0.9584	33.933	0.9479

**Table 8:** The average PSNR and SSIM results of different algorithms in Lung-PET-CT-Dx dataset with different scaling factors.

Model	×2		×3		×4	
	PSNR	SSIM	PSNR	SSIM	PSNR	SSIM
Bicubic	24.990	0.8404	24.042	0.7994	23.163	0.7620
SRCNN	38.503	0.9814	33.622	0.9530	30.844	0.9262
FSRCNN	38.688	0.9818	34.025	0.9544	31.157	0.9280
VDSR	40.656	0.9841	35.773	0.9612	31.966	0.9375
DRRN	40.352	0.9839	35.858	0.9615	32.858	0.9408
EDSR	40.975	0.9845	36.455	0.9628	33.569	0.9436
MDSR	41.030	0.9846	36.456	0.9627	33.472	0.9430
RDN	41.159	0.9848	36.792	0.9638	33.882	0.9453
RCAN	41.222	0.9849	36.887	0.9641	34.198	0.9463
IDMAN (ours)	41.319	0.9850	36.981	0.9644	34.253	0.9465

## Data Availability

The datasets used in this paper are public datasets. The DeepLesion could be found from https://nihcc.box.com/v/DeepLesion (accessed on 29 September 2021). The NSCLC Radiogenomics could be found from http://doi.org/10.7937/K9/TCIA.2017.7hs46erv (accessed on 29 September 2021). The Lung-PET-CT-Dx could be found from https://doi.org/10.7937/TCIA.2020.NNC2-0461 (accessed on 29 September 2021).
